# Assessing governance structures for climate-resilient and sustainable health systems: development of the PHONIC framework

**DOI:** 10.7189/jogh.16.04083

**Published:** 2026-05-29

**Authors:** Karin Geffert, Annkathrin von der Haar, Marie Jung, Melvine Anyango Otieno, Eva Rehfuess, Alexandra Schneider, Maike Voss, Isabelle von Polenz, Franziska Matthies-Wiesler

**Affiliations:** 1 Institute of Medical Information Processing, Biometry and Epidemiology (IBE), LMU Medizin, Ludwig-Maximilians-Universität München, Munich, Germany; 2 Pettenkofer School of Public Health, Munich, Germany; 3 Centre for Planetary Health Policy, Berlin, Germany; 4 Planetary Health Eastern Africa Hub, Eldoret, Kenya; 5 School of Environmental Sciences and Natural Resource Management, University of Eldoret, Eldoret, Kenya; 6 Global and Planetary Health Working Group, Institute for Medical Epidemiology, Biometrics and Informatics, Martin-Luther-University Halle-Wittenberg, Halle (Saale), Germany; 7 Institute of Epidemiology, Helmholtz Zentrum München – German Research Center for Environmental Health (GmbH), Neuherberg, Germany; 8 neues handeln AG, Berlin, Germany; 9 Department of Nuclear Medicine, LMU University Hospital, LMU Munich, Munich, Germany

## Abstract

**Background:**

The consequences of climate change have detrimental effects on human health. The establishment of functional governance structures is considered crucial for addressing health challenges, as such structures provide the foundation for coordinated and coherent action. At present, no standardised and internationally applicable approach exists for assessing and benchmarking governance structures for climate and health at national level. Therefore, the objective of this study was to develop the PHONIC framework, a tool designed to assess governance structures for climate-resilient and sustainable health systems.

**Methods:**

We followed a four-step approach: First, we selected a methodology, originally designed for assessing food environment policies, and assessed its applicability to governance structures for health and climate change. This methodology comprises five key stages, as well as a set of indicators and a catalogue of good practice examples. Second, we identified and adapted existing indicators, through online consultations with international experts, and collated good practice examples for the assessment of governance structures for climate-resilient and sustainable health systems. Third, we piloted the framework with national expert groups in two countries, Germany and Kenya. Finally, we evaluated the PHONIC framework’s application and revised the methodology based on the findings from the pilot phase.

**Results:**

The final PHONIC framework includes a set of ten indicators across three thematic areas: three indicators focused on governance, four on policy development, and three on cross-sectoral collaboration. In addition, we compiled a catalogue of good practice examples for climate-resilient and sustainable health systems, currently comprising 47 examples from 32 countries. The piloting revealed opportunities, such as the participatory process, but also challenges regarding the narrow focus on health systems.

**Conclusions:**

The PHONIC framework, with its participatory and adaptable approach, enables the benchmarking and comprehensive analysis of the strengths and weaknesses of governance structures for climate-resilient and sustainable health systems at country level. The potential of the framework could be expanded by including international experts from a broader geographic scope and systematic searches for good practice examples. Decision makers in the health sector can use the outcomes of the PHONIC framework to identify the most relevant areas and actions for improvement of their climate change and health governance.

Climate change poses a global threat to human health both through direct and indirect pathways, exacerbated by vulnerability factors [1,2]. Health impacts include mortality, injuries, and increased disease burden due to extreme weather events as well as shifts in the incidence of infectious diseases. Additionally, climate change has significant effects on mental and psychosocial health. Beyond these direct impacts, it also affects a broad range of social determinants of health such as livelihoods and the functioning of health systems [2]. Addressing these complex and interconnected health challenges requires functional governance structures which serve as the foundation for coherent actions across sectors [3]. Such structures support essential functions including policy development, resource stewardship, evaluation and monitoring, engagement of partner institutions, exercising legal authority and provision of leadership [4]. Assessing governance structures is therefore critical for identifying gaps and opportunities for strengthening resilience and for enabling policymakers to design more integrated and climate-resilient health systems [5].

There is a high interest from policy and practice to understand strengths and weaknesses of existing governance systems. Multiple frameworks for evaluating and assessing governance of health systems with varying underlying theories and methodologies originating from different disciplines exist. A systematic review found that most identified frameworks to assess health-systems governance are based on qualitative methods, stating that governance is the outcome of the interaction between different actors and therefore a valuable measure for the assessment of governance systems [6]. The World Health Organization (WHO) Health system performance assessment is a key health-system assessment tool, but does not specifically focus on aspects of climate change [7]. Also of importance is the question of the implementing sector, which may be research, policy, practice or a development partner [7]. In ecological sciences, there are also various governance frameworks available which include aspects of complexity theory and that are based on social, policy, and ecological sciences [8], but do not include health perspectives.

While the variety of governance evaluation frameworks and methodologies prove the interest of research and practice alike in this topic, there are also challenges in applying these frameworks and methods with limited resources at national level for actors outside the government system.

A range of the existing health-system governance frameworks have been selectively applied in low-middle income countries (LMIC). Still, they are largely not climate-specific and often emphasise structural performance metrics over participatory or cross-sectoral processes, making cross-country comparison difficult [6,9,10]. In Africa and other LMIC regions, benchmarking tools such as the African Union health financing scorecards illustrate measurement of system performance, yet focus on inputs/expenditure rather than governance processes [11,12]. Evidence from climate-health and adaptation studies in LMICs consistently highlights the centrality of political will, intersectoral collaboration, and institutional capacity for effective action [13,14]. Together, this indicates a gap for a standardised, adaptable framework enabling cross-country comparison of governance specifically for climate-resilient health systems.

To the best of our knowledge, there is currently no standardised and widely accepted method for assessing and benchmarking governance structures for climate change and health at the national level across countries. While several benchmarking and accountability initiatives from scientific and non-profit institutions exist, such as the Lancet Countdown on Health and Climate Change [15] and the Healthy National Determined Contribution (NDC) Scorecard developed by the Global Climate & Health Alliance [16], these primarily focus on broader health and climate indicators rather than on governance structures specifically.

A major challenge lies in the use of the term ‘governance,’ which, despite its frequent application, varies significantly in definition and scope depending on the context [6]. This conceptual ambiguity complicates the development of consistent assessment frameworks and makes cross-country comparisons difficult.

Therefore, the objective of this study was to develop a framework to assess governance structures for climate-resilient and sustainable health systems for actors outside of government systems. The specific objectives were to:

1) select an existing methodology and assess its applicability to governance for climate-resilient and sustainable health systems,

2) develop a set of indicators and compile a catalogue of good practice examples,

3) pilot the draft methodology with national expert groups in Germany and Kenya, and

4) evaluate the pilot application and advance the methodology accordingly.

## METHODS

This study is embedded in the Public Health OperatioNs for clImate aCtion (PHONIC) project. PHONIC aims at assessing and prioritising public health operations for climate change mitigation and adaptation. It was implemented by an interdisciplinary research team comprising members from universities and non-governmental organisations (NGO) in Germany and Kenya with expertise in climate-resilient and sustainable health systems. An international advisory board accompanied the project and was consulted on a biannual basis for feedback.

**Figure 1:**
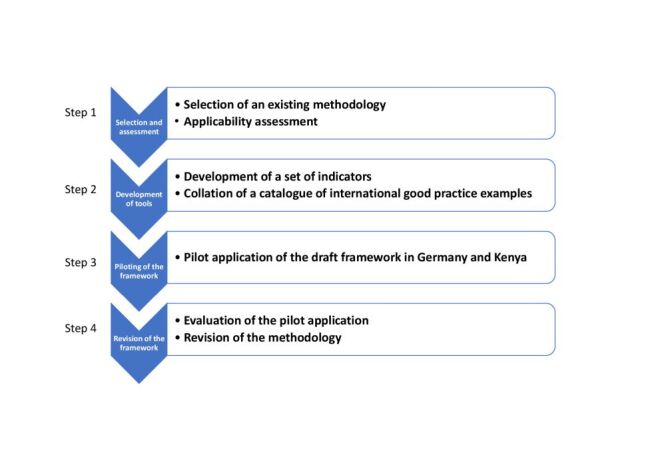
Development of the PHONIC framework for assessing governance structures for climate-resilient and sustainable health systems.

We followed four main steps (**Figure 1**).

### Step 1. Selection and applicability assessment of an existing methodology

For this study, we decided to use the process of policy benchmarking, defined as ‘a continuous process of comparing, identifying, and adapting best practice with the aim of improving performance’ [6]. Benchmarking is considered a tool for steering of political agendas and policy learning [17] and involves the use of comparative metrics to assess performance of different systems [18]. This is done by breaking down complex phenomena into numeric values. In the context of public health, benchmarking of processes, policies, and strategies serves multiple purposes: assisting in recognising top performers, promoting competition, enhancing stakeholder engagement, and fostering a shared understanding among key actors. Moreover, the systematic collection of benchmarking data over time enables policy evaluation and trend analysis. In addition, performance analysis can help uncover barriers and facilitators to policy implementation, supporting more informed and effective decision-making [19].

As there was no scientifically established benchmarking methodology in the field of health and climate, we selected the Healthy Food Environment Policy Index (Food-EPI), an international gold standard for assessing government action for healthy food environments for the overall methodological approach [20]. This methodology was chosen due to its systematic approach, which has been applied and scientifically evaluated in over 40 countries. Moreover, the structured process of comparing and benchmarking policy actions has demonstrated its utility across various contexts and proved to be manageable in terms of the resources required.

The Food-EPI consists of different stages, including:

1) setting up an expert group,

2) adapting a set of indicators to the national context,

3) conducting a situation analysis,

4) benchmarking against international good practice examples, and

5) identifying and prioritising improvement actions [20].

The Food-EPI is designed to be flexible, allowing for adaptation to different national contexts by modifying or omitting certain indicators as necessary. In a series of research team meetings, we systematically reviewed the Food-EPI process to assess its suitability and determine the necessary adaptation requirements for addressing governance structures in the context of climate-resilient and sustainable health systems.

### Step 2. Development of a set of indicators and a catalogue of good practice examples

In 2023, the WHO published an operational framework for building climate-resilient and low carbon health systems [21]. This framework outlines indicators across ten key components. The first component, climate-transformative leadership and governance, focuses on governance, policy development, and cross-sectoral collaboration. It forms the essential foundation for successful and effective steering, management, and implementation of climate-resilient health systems. Despite being rather vague in nature and the difficulty of measuring ‘good governance’, governance measures are of key importance for progress. Given its relevance, this component therefore served as the starting point for the development of indicators and the collection of good practice examples for the PHONIC framework.

#### Step 2.1. Development of the set of indicators

The component on climate-transformative leadership and governance of the WHO operational framework consists of a total of 18 indicators: six related to governance, seven to policy development and five to cross-sectoral collaboration [21]. This indicator set was scrutinised by the research team to appraise its applicability to assessing governance structures for climate-resilient and sustainable health systems.

Adaptations of the indicators were carried out through two online consultations with a group of 13 international experts. These experts were identified through purposive sampling aiming to include representatives from health authorities, academia, research, NGOs and community networks. The participating experts brought expertise from different fields including public health, medicine, law, environmental management, sustainability and climate risks, and were based in Germany (five experts), Kenya (seven experts) and Finland (one expert).

These consultations, conducted between January and March 2024, lasted 60–90 minutes each. Their objectives were to review and amend the set of indicators and to discuss criteria for prioritising them. A first set of indicators based on the WHO framework were presented to the groups and experts were asked to discuss their applicability and relevance. The number of indicators was reduced from 18 to 10 on the basis of relevance and practicability. After the consultations, the selected and adapted indicators were shared with the experts for further review. As a result of these discussions, the indicator set was refined to improve methodological applicability. Indicators scope and wording were adapted for the remaining indicators based on three main criteria: specificity, meaningfulness, and measurability. The revised indicators underwent further re-evaluation through an iterative process involving the international experts, the research team, and feedback from the PHONIC project’s advisory board. This process led to the final indicator set used in the PHONIC framework.

#### Step 2.2. Collation of the catalogue of good practice examples

To enable a qualitative assessment of indicator implementation against a standard, a set of international good practice examples were compiled for each of the indicators developed in Step 2.1. The aim was to create a globally relevant catalogue of good practice examples that offers a broad representation of different contexts, ensuring applicability across diverse national settings. The initial selection of good practice examples was identified through a scoping review [22]. Additional good practice examples were extracted from a review on health-related activities in national climate change adaptation plans [23], as well as from a review on health representation in nationally determined contributions (NDCs) and long-term strategies [24]. Following a snowball approach, websites of selected international and national health authorities were reviewed to gather more detailed information and relevant publications. To further supplement this collection of good practice examples, targeted literature searches to support the ten defined indicators were conducted using Google and Google Scholar. For each of the indicators, relevant combinations of search terms were extracted from the description, the actual wording of the respective indicator. For example, for indicator 1.3.1 the search terms such as ‘vulnerabilities’, ‘health inequities’, ‘health’, and ‘climate change’ and ‘strategies’ were combined to identify appropriate documents describing suitable examples in relation to this indicator 1.3.1. A new set of search terms was constructed for each of the ten indicators in analogy.

The resulting list of examples encompassed activities at national or regional level, specifically related to governance, policy development, and cross-sectoral collaboration. Each example was reviewed and discussed within the research team. Inclusion or exclusion decisions were based on the following several criteria: the example's relevance to the corresponding indicator, its suitability as a model of good practice, the availability of concrete information, and the need to ensure a balanced representation of geographical and contextual diversity. The final set of good practice examples were compiled into an Excel file hereafter referred to as ‘the catalogue’. The catalogue is not considered a rigid document, but rather one to which new documents can be added as the processes move on and more initiatives and strategies evolve and are being developed.

### Step 3. Piloting of the draft framework in Germany and Kenya

The PHONIC framework was piloted in Germany and Kenya with the aim of identifying strengths and weaknesses in practical application of the draft framework. These two countries were chosen due to their contrasting contexts: they differ in the organisation of their health systems (federalist in Germany *vs*. centralised in Kenya), their vulnerabilities to climate change exposure, the nature and extent of current and projected climate change-related health impacts, and their prior experience with regard to implementing climate-resilient and sustainable health systems strategies [25–27].

The piloting process followed the same steps as the Food-EPI methodology. This included the set-up of national expert groups, adaptation of the indicators to the country context, semi-systematic assessment of the national status quo, benchmarking against selected good practice examples from the catalogue, and prioritisation of improvement actions. Due to feasibility constraints, the final step, prioritisation of improvement action was conducted only in Germany. The benchmarking involves a five-point likert scale to assess implementation (0–20%, 20–40%, 40–60%, 60–80%, 80–100%) which is completed by participating experts for each indicator. Then, the mean of the responses is calculated to assess the average rating.

The detailed processes and results of the piloting are described elsewhere (manuscript under review). In this article we present the feedback from the two national expert groups on the PHONIC framework. Input was collated during half-day online workshops conducted in Kenya and Germany in May 2024. The workshops aimed to present the methodological process, validate the results of the situation analysis, and review the benchmarking outcomes.

In Germany, the national expert group was also invited to provide written feedback on the indicators during the benchmarking survey. Additional observations were collated during a one-hour virtual meeting in August 2025 with the aim of presenting the outcomes of the benchmarking exercise and discussing improvement actions for enhancing governance structures.

### Step 4. Evaluation of the pilot application and revision of the framework

To iteratively refine the methodology of the PHONIC framework, the research team took records of general observations throughout the piloting process, including noted challenges and opportunities. Additional insights were gathered from individual discussions with members of the national expert groups. These observations and reflections were systematically reported and discussed during biweekly meetings, where the team collaboratively identified areas requiring adjustment. Based on these discussions, the PHONIC framework and its methodology were continuously adapted accordingly. The reporting of the framework development is based on the Good Reporting of A Mixed Methods Study (GRAMMS) checklist (**Online Supplementary Document**) [28].

## RESULTS

### Step 1. Selection and applicability of the Food-EPI approach

Based on a first assessment, the Food-EPI approach was considered relevant to the context of health and climate change governance, with no major changes needed. More in-depth reflection revealed the necessity for adapting the methodological tools, *i.e*. indicators for health and climate change operations and corresponding international good practice examples required for adaptation to ensure contextual relevance and applicability.

### Step 2. The PHONIC framework’s indicators and catalogue of good practice examples

#### Step 2.1. The set of indicators

A set of indicators was developed to assess governance structures necessary for strengthening the resilience of health systems towards climate change impacts (**Table 1**). The final set consists of 10 indicators, allocated to three thematic areas. Two of these indicators are further differentiated into sub-indicators to capture more specific dimensions of governance performance. The indicator set is designed to be adaptable to various contexts across countries or at governance levels within a single country, ideally through expert consultation.

**Table 1:** Indicators to assess governance structures for climate-resilient and sustainable health systems adapted from WHO operational framework [19]

Objectives	Indicator
Health governance for climate change and health (1)	1.1. A person or team working on climate change and health is designated within the health ministry and the responsible health agency (at national and subnational level)
	1.2. Teams or units for climate change and health (at national and subnational level) designated within ministries or responsible agencies are working in collaboration with relevant climate-sensitive health programmes (*e.g*. monitoring and surveillance on vector-borne diseases, heat).
	1.3.1 Vulnerabilities and health inequalities/inequities are streamlined, explicitly named and defined in the regulations and strategies on climate change and health.
	1.3.2 If applicable: Vulnerability and adaptation assessments (V&As) are repeatedly conducted, publicly reported and serve as a basis for improvement and development of (new) mitigation and adaptation policies and programmes in health-determining sectors.
Policy development for climate change and health (2)	2.1.1. A national strategy/roadmap on health and climate change is developed, considering both resilience and low-carbon sustainability approaches with cross-sectoral collaboration (governmental and non-governmental actors).
	2.1.2 If applicable: The given national strategy/roadmap is implemented (with corresponding regulations).
	2.2.1 Health sector commitment with clearly defined goals to transition the health system (including health care facilities and supply chains) to low carbon or net-zero emissions.
	2.2.2 If applicable: Mechanisms to estimate, monitor and regularly report greenhouse gas emissions and the goal achievements in the health system are established.
	2.3. Commitment by the government is made to use evidence for health and climate change decision-making processes and to strengthen further evidence generating.
	2.4 Multi-stakeholder engagement activities (at the national and subnational level, *e.g.* consultation workshops, roundtables) are explicitly undertaken to inform the development and adaptation of climate change and health strategies and regulations.
Cross-sectoral collaboration for climate change and health (3)	3.1. Regular exchange platforms are established between the Ministry of Health and key stakeholders (*e.g*. governmental agencies, research institutions, civil society, private sector) at the national level (if applicable also at the sub-national level).
	3.2. Inter-ministerial and/or inter-agency working groups on climate change and health are established with regular working meetings, promoting health in all adaptation and mitigation policies of key health-determining sectors.
	3.3.1 Health and environmental impact assessments are being conducted and regularly reported.
	3.3.2 If applicable: The results of these assessments are used for developing, adapting and implementing (new) mitigation and adaptation policies and programmes in health-determining sectors.

#### Step 2.2. The catalogue of international good practice examples

At time of publication, the catalogue contained information on 47 examples from 32 countries (data not yet published). The collection is organised according to the indicator sets for governance (14 examples), policy development (17 examples) and cross-sectoral collaboration (16 examples), whereby certain examples may appear under more than one indicator. The design allows for sorting and filtering by indicator, practice example, continent and country to facilitate targeted searches. For each good practice example, a short description is given and a link to further information is provided.

### Step 3. Experience with the PHONIC framework in Germany and Kenya

Members of the national expert groups reported opportunities as well as challenges regarding the piloting of the PHONIC framework. They underlined opportunities linked with the participatory process such as exchange and networking with experts in the field, a comprehensive overview of governance structures provided by the evidence report, learnings from implementing an international framework at the national level, and identifying areas for improvement through the benchmarking process. It was also noted that the differentiation made between existing plans and strategies *vs*. their actual implementation allowed for a more nuanced analysis.

The main challenge regarding the piloted methodology was the narrow focus of the indicator set on health systems rather than on other political sectors. It was repeatedly mentioned that climate change and its interaction with health is a cross-sectoral topic which requires analysis of further sectors such as economy, transport, energy, housing and social welfare. Experts also highlighted that a major shortcoming of the currently implemented policy actions lies in the lack of or insufficient collaboration between these sectors. In their opinion, measures that only address singular sectors would be insufficient to tackle the complex and interconnected challenges posed by climate change. Instead, they stressed the need for integrated, cross-sectoral approaches.

Further challenges were raised regarding the identification of good practice examples. Experts noted the difficulty in reaching consensus on what constitutes ‘good’ governance structures, as definitions and evaluation criteria vary across contexts. Lastly, gaps between policy ambitions and the existence of certain functionalities of governance structures were repeatedly mentioned, as well as a lack of effective implementation of existing measures.

### Step 4. Evaluation of the pilot application and revision of the PHONIC framework

The results of piloting the PHONIC framework in Germany and Kenya are in detail presented in a different paper (manuscript under review elsewhere). Briefly summarised, in Germany, health ministry and agency teams working on climate change and inter-agency collaboration were rated as highly implemented, while monitoring mechanisms and health-environment policy assessments received lower scores, with implementation consistently lagging. In Kenya, top ratings went to ministry teams and national cross-sectoral strategies, but commitments to low-carbon transitions, evidence use, and impact assessments scored lower, again revealing an implementation gap across both countries.

Discussions within the research team on applying the PHONIC framework in the two contexts revealed three main observations.

First, the participatory approach for applying the PHONIC framework required a high level of resources but was also perceived to be of significant added value. The national expert groups needed to prepare themselves extensively to acquire detailed knowledge of their country-specific structures of relevance for health and climate change. This was particularly challenging as not all information was publicly available, and as the topic area was not yet as well explored as other policy domains. However, the participatory approach provided a valuable platform for experts from different disciplines and sectors to talk about and reflect on common challenges, build a mutual understanding and learn from each other’s perspectives and experiences. The level of engagement of the national expert group was high, both during discussion rounds and individual exchanges with the research team, attesting to their interest in the methodological approach and its outcome. We therefore concluded that the participatory approach of the PHONIC framework should be retained.

Second, we observed a tension between the wish for a very comprehensive approach and the need to keep the assessment of governance structures feasible. The indicator sets’ focus on health systems and their measures to address climate change could only partially reflect activities in other relevant sectors. However, this defined focus kept the process of implementing the PHONIC framework manageable, avoided an overburdening of the experts and enabled a more granular comparison of relevant indicator aspects. Experts also agreed on having indicators that are precise, measure fewer dimensions instead of expanding them and integrating more topics. In conclusion, the designation of the PHONIC framework was confirmed: instead of assessing all governance structures for climate change and health, we maintained a concentration on climate-resilient and sustainable health systems.

Third, it was noted that there was limited consensus within members of the national expert groups regarding the actual impact of improved governance structures for climate-resilient and sustainable health systems. This observation reveals a general need for agreement on concrete action towards ‘good’ governance structures for climate-resilient and sustainable health systems. After piloting the PHONIC framework, we concluded that the above-mentioned participatory elements, despite their need for significant resources, are crucial for developing a shared understanding and agreement on the elements of ‘good’ governance structures.

## DISCUSSION

We designed, tested, and evaluated a multi-stage approach for assessing governance structures that support climate-resilient and sustainable health systems [21]. The PHONIC framework is rooted in the WHO operational framework for building climate-resilient and low carbon health systems. Its development process involved a multi-disciplinary research team, and was informed by existing indicators, good practice examples, and input from international experts, who played a central role in refining and advancing the framework’s content. Subsequent piloting in Germany and Kenya showed that applying the PHONIC framework is feasible and produces insights that are considered relevant and meaningful by national experts. It also revealed both opportunities and challenges with regards to implementing the PHONIC framework. To begin with, assessing the topic of governance for climate change and health has proven to be a challenging task. While the term ‘governance’ is commonly used, its meaning can vary in different contexts. According to the definition of the WHO from 2007 ‘governance is ensuring (that) strategic policy frameworks exist and are combined with effective oversight, coalition-building, regulation, attention to system design and accountability’ [29]. Another definition describes health governance as ‘overlapping and sometimes competing (governance) regime clusters that involve multiple players addressing different problems through diverse principles and processes’ [30]. In the specific context of climate change, governance is described as ‘complicated and challenging given the necessary involvement of multiple sectors and scales, including the increasing activity of actors “beyond the state” such as non-government and private organisations’ [31]. These definitions have in common that governance captures both processes and structures, including multiple institutions and actors. A review on ‘climate-resilient development’ of health care systems did not identify evidence on the role of governance, but instead identified several studies addressing barriers to good governance, such as financial constraints, uncertainty, leadership issues, and a lack of knowledge [3]. The authors conclude that climate-resilient governance is closely tied to actors, institutions, and networks, yet notably, no study identified governance as an enabler.

While governance has been discussed in several specific disciplines, there is yet no unified definition of governance structures for addressing climate change in the context of health systems. Despite this fact, certain elements or sub-functions of governance of health systems are widely agreed upon, including policy and vision, stakeholder voice, information and intelligence, legislation, and regulation. The operationalisation of these areas is influenced by the viewpoint and the access to information of the actors implementing a policy analysis framework [7]. The PHONIC framework is therefore designed to be used by actors outside the government system, but the implementation should be done in close collaboration and consultation with actors from within the government system. With its focus on national processes and outcomes, the outcomes of the PHONIC framework are most effective in national policy-making and identification of areas for improvement. Country-comparison of the outcomes is in general possible, however, due to the differences in implementing actors, national expert groups and health systems limited in generalisability. In addition, the outcomes could, in theory, support official reporting mechanisms for the Sustainable Development Goals or the National Adaptation Plans under the United Nations Framework Convention on Climate Change.

The governance structures addressed by the PHONIC framework focus primarily on health systems and related government actions. While this is limited in scope, it enables a more detailed analysis of these aspects. We acknowledge that other methodological approaches can be applied to assess governance structures and that broader perspectives might be better suited to gain an overview of the whole system of governance across multiple sectors. However, we believe that the approach proposed here, which is based on the WHO operational framework [21], adds value as it is specifically tailored to experts in the (public) health sector. Based on the piloting experience, we recommend setting a clear and narrow focus for analyses to ensure a feasible process, while also recognising and discussing the limitations and possible pitfalls that this might entail. Similarly, out of the ten components in the original WHO operational framework for building climate-resilient and low carbon health systems, the PHONIC framework focused only on governance. In the research team’s view, governance represents the core component for the implementation of all other components of the WHO framework.

The PHONIC framework, in its current version and with its established set of indicators and good practice examples, can, in principle, be applied in different countries. The staged approach allows for a country-specific assembly of national experts and for national and even sub-national adaptation of indicators. For this, the indicators of the PHONIC framework should be reviewed by (sub-)national experts and checked for their applicability and/or need for adaptation to the given context. Due to the iterative approach of the adaptation process, we do not propose weighting the indicators as this would likely differ between contexts. Further applications could also contribute to enhancing the current catalogue of good practice examples, thereby enhancing the evidence base across different countries and health systems. The application of the PHONIC framework is, however, limited to countries with stable government systems and relies on a certain level of transparency in policy-making processes for creating the evidence report and getting a realistic perspective of the situation in the country.

There is also further potential for advancing and expanding the PHONIC framework. The methodology presented here could be applied to all other components and functions of the WHO operational framework [21]. Potentially, when repeated with recurring expert groups and indicators, our methodology might also allow tracking and comparison of changes over time, *e.g*. progress or regression of governance structures.

However, there are limitations in relation to both the development and the application of the PHONIC framework. The good practice examples used for benchmarking were collected through a semi-systematic process, whereas more comprehensive and systematic searches in scientific and grey literature would likely have yielded more results. Even with a large amount of data, it remains challenging to identify a benchmarking standard against which to compare what can be considered ‘good’, especially regarding governance and its implementation. While national processes of adapting the indicators, compiling evidence and assessing the degree of national implementation were innovative and informative, they were also challenging. It became clear that the lack of clear evidence on 'ideal solutions' may hinder the effective use of numeric scales for assessing implementation, potentially making the process feel challenging and insufficient. In this respect, this PHONIC framework and accompanying methodology relies on a common set of underlying norms and values of participating experts, for example regarding the role of the state in addressing climate change-related health challenges and the usefulness of transparent and collaborative policy approaches. For this reason, the selection of national experts should be representative of a broad range of different actors, in an attempt to reduce potential bias, for example, when certain sectors are overrepresented. Further, for reasons of feasibility, our study was undertaken with a limited number of international experts thus potentially introducing bias. In addition, the piloting focussed on two countries and lacked variety in terms of geographic scope, disciplines and sectors. Nevertheless, the exploratory nature of this project had allowed us to test an innovative methodology enabling collaborative assessments of complex issues as well as the creation of a good practice catalogue, which can be used in future assessments. Further uptake of the findings from implementing the PHONIC framework could be considered for the work of the WHO framework for low-carbon and climate-resilient health systems.

## CONCLUSIONS

The PHONIC framework provides a structured foundation for assessing and benchmarking governance structures for climate-resilient and sustainable health systems in a standardised, comprehensive, and participatory manner. Its flexible methodology allows for adaptation to various national or sub-national contexts, notably through the identification and engagement of national experts, the context-specific selection of indicators, and the tailored use of good practice examples from the catalogue. As next steps, the PHONIC framework should be validated in further countries from different contexts in order to assess its further applicability. The outcomes of the PHONIC framework are intended to inform primarily national policy making but has the potential to support also international policy processes.

## Additional material


Online Supplementary Document
