# Supplementary material for: Assessing governance structures for climate-resilient and sustainable health systems: development of the PHONIC framework
Source: J Glob Health. 2026 May 29;16:04083. doi: 10.7189/jogh.16.04083 (PMC13220648; doi:10.7189/jogh.16.04083)
Supplement: Online Supplementary Document [file jogh-16-04083-s001.pdf]

Supplement to: Geffert K, von der Haar A, Jung M, Otieno MA, Rehfuess E, Schneider A, Voss M, von Polenz I, Matthies-Wiesler F. Assessing governance structures for climate-resilient and sustainable health systems: development of the PHONIC framework. J Glob Health. 2026;16:04083.

Good Reporting of A Mixed Methods Study (GRAMMS) checklist

Guideline	Section: page
Describe the justification for using a mixed methods approach to the research question	Methods: p. 4
Describe the design in terms of the purpose, priority and sequence of methods	Methods: p. 4 following
Describe each method in terms of sampling, data collection and analysis	Methods: p. 4 following
Describe where integration has occurred, how it has occurred and who has participated in it	Methods, Step 4: p. 6
Describe any limitation of one method associated with the present of the other method	Discussion, p. 11
Describe any insights gained from mixing or integrating methods	Discussion, p. 11

O'Cathain A, Murphy E, Nicholl J. The quality of mixed methods studies in health services research. J Health Serv Res Policy. 2008;13: 92-98.

Text S1. Explanation of authorship change statement

One of our co-authors, Annkathrin von der Haar, was unavailable during the manuscript submission process. As Annkathrin von der Haar was deeply involved in the conduction of the project, and has now rejoined the process, we would like to acknowledge her contribution accordingly through authorship.
